# Integrated Microfluidic Preconcentration and Nucleic Amplification System for Detection of Influenza A Virus H1N1 in Saliva

**DOI:** 10.3390/mi11020203

**Published:** 2020-02-16

**Authors:** Yonghee Kim, Abdurhaman Teyib Abafogi, Buu Minh Tran, Jaewon Kim, Jinyeop Lee, Zhenzhong Chen, Pan Kee Bae, Kyoungsook Park, Yong-Beom Shin, Danny van Noort, Nae Yoon Lee, Sungsu Park

**Affiliations:** 1 School of Mechanical Engineering, Sungkyunkwan University, Suwon 16419, Korea; hanskim723@gmail.com (Y.K.); ab18aa@gmail.com (A.T.A.); jaewon1394@gmail.com (J.K.); softmemsljy@naver.com (J.L.); journey12580@gmail.com (Z.C.); 2 Department of BioNano Technology, College of BioNano Technology, Gachon University, Seongnam 13120, Korea; tbminh420@gmail.com (B.M.T.); nylee@gachon.ac.kr (N.Y.L.); 3 BioNano Health Guard Research Center (H-GUARD), Daejeon 34141, Korea; bpkee@kribb.re.kr (P.K.B.); marsp@kribb.re.kr (K.P.); ybshin@kribb.re.kr (Y.-B.S.); 4 Bionanotechnology Research Center, Korea Research Institute of Bioscience and Biotechnology (KRIBB), Daejeon 34141, Korea; 5 Department of bioengineering, KRIBB School, University of science and Technology (UST), Daejeon 34141, Korea; 6 Department of Physics, Chemistry and Biology, Linköping University, 581 83 Linköping, Sweden; 7 Chair of Micro Process Engineering and Technology (COMPETE), University of Ljubljana, 1000 Ljubljana, Slovenia; 8 Centro de Investigación en Bioingeniería -BIO, Universidad de Ingenieria y Tecnologia—UTEC, Barranco 15036, Peru; 9 Biomedical Institute for Convergence at SKKU (BICS), Sungkyunkwan University, Suwon 16419, Korea

**Keywords:** microfluidic device, immunomagnetic separation, molecular amplification, H1N1

## Abstract

Influenza A viruses are often present in environmental and clinical samples at concentrations below the limit of detection (LOD) of molecular diagnostics. Here we report an integrated microfluidic preconcentration and nucleic amplification system (μFPNAS) which enables both preconcentration of influenza A virus H1N1 (H1N1) and amplification of its viral RNA, thereby lowering LOD for H1N1. H1N1 virus particles were first magnetically preconcentrated using magnetic nanoparticles conjugated with an antibody specific for the virus. Their isolated RNA was amplified to cDNA through thermocycling in a trapezoidal chamber of the μFPNAS. A detection limit as low as 100 TCID50 (50% tissue culture infective dose) in saliva can be obtained within 2 hours. These results suggest that the LOD of molecular diagnostics for virus can be lowered by systematically combining immunomagnetic separation and reverse transcriptase-polymerase chain reaction (RT-PCR) in one microfluidic device.

## 1. Introduction

The influenza virus causes acute infectious diseases in the human respiratory tract [1,2,3,4,5]. The World Health Organization (WHO) has estimated over 1 billion cases of influenza globally each year. Unless it is detected at an early stage and proper medication is applied [3,6], it can easily spread and cause an influenza outbreak [3] with higher transmissibility [4]. Various diagnostic methods, such as virus isolation [5,7,8,9,10], enzyme-linked immunosorbent assay (ELISA) [5,8,9,10], and reverse transcriptase-polymerase chain reaction (RT-PCR) [7,8,9,10,11] are used to detect influenza virus. RT-PCR is the most widely used method for the amplification of microbial genes, due to its accuracy and technical maturity [9,12,13]. However, its use in clinical samples such as saliva often fails to obtain sensitive results due to the presence of impurities that interfere with PCR [14,15,16,17,18]. Previous reports showed that saliva inhibited PCR and cumbersome sample preparation steps were required to extract the viral genome [17,18]. 

Sample preparation methods, such as preconcentration and nucleic acid purification of pathogens, are required for the amplification of target genes of the pathogens in clinical samples [16,19,20,21,22,23,24]. Thus, various sample preparation methods, including immunomagnetic separation (IMS) using antibody-conjugated magnetic nanoparticles (Ab-MNPs), were used to preconcentrate pathogens and cancer cells in order to improve the sensitivity of PCR and immunoassays [21,25,26]. However, these methods usually rely on manual operation, which is very time consuming and requires highly skilled personnel with a variety of equipment and dedicated laboratory space [23,24]. Because of these drawbacks, they are not considered suitable for use in molecular diagnostics where either rapid detection is required, or modern laboratory facilities are remote or absent [27]. For detection in these circumstances, simple and easy sample preparation techniques should be developed. 

In this regard, a microfluidic device (μFD) is considered an excellent platform for employing the sample preparation methods due to its high speed and ease of integration and automation [26,28,29,30,31,32]. Previously, we demonstrated that the limit of detection (LOD) for a bacterial pathogen in a large volume of samples can be improved by combining IMS with PCR in a μFD [20]. This improvement was possible due to the fact that the high surface area of the μFD allows pathogens and Ab-MNPs to rapidly interact with each other [33], while the narrow channel of the μFD allows Ab-MNPs to be easily and quickly captured to the permanent magnet underneath the channel [20]. 

**Figure 1:**
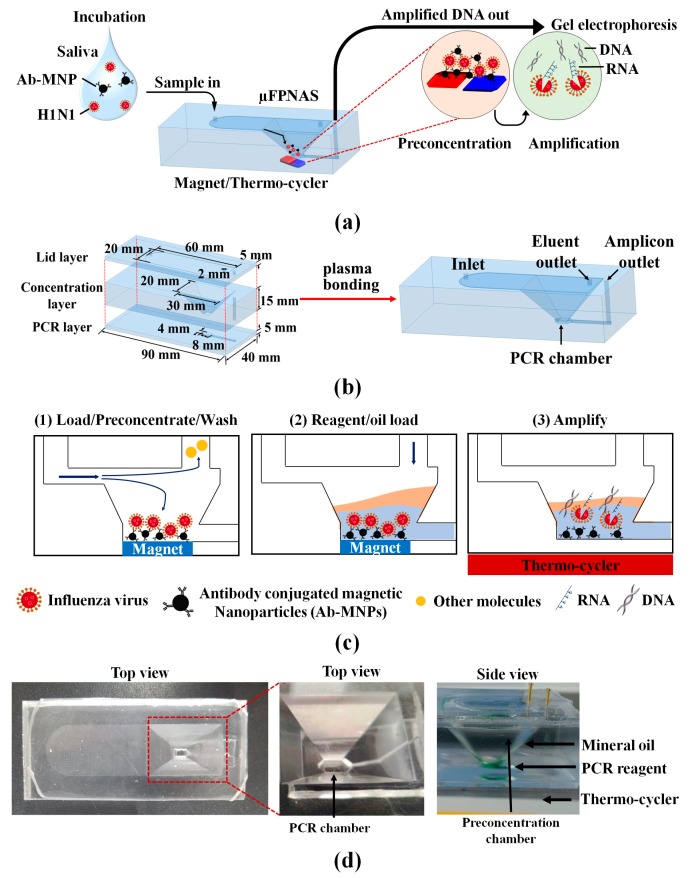
Design of microfluidic preconcentration and nucleic amplification system (µFPNAS) and its operation. (**a**) Its schematic describing on-chip preconcentration and nucleic acid amplification. (**b**) Its layers (top: lid, middle: concentration, bottom: polymerase chain reaction (PCR)), two chambers (preconcentration and PCR), and two inlets and two outlets showing their respective dimensions. (**c**) A schematic describing its operation protocol. (**d**) Images of µFPNAS mounted on a on a heat block (10 cm × 10 cm) modified from C1000 Touch™ thermocycler (Bio-RAD, Hercules, CA, USA).

In this study, we report a microfluidic preconcentration and nucleic amplification system (μFPNAS) that enhances the sensitivity of PCR-based detection of influenza A virus (H1N1) by first preconcentrating H1N1 virus particles in saliva with Ab-MNPs and then amplifying their RNA. It consists of a trapezoidal preconcentration chamber, a microchamber, two inlets and two outlets. RNA of concentrated H1N1 virus was amplified by RT-PCR using a thermo cycler placed under the µFPNAS (Figure 1a).

## 2. Materials and Methods

### 2.1. Influenza A Virus (H1N1) and Its Titration

H1N1 strain (A/California/04/2009) and anti-H1N1 antibody were provided by the Korea Research Institute of Bioscience and Biotechnology (KRIBB, Daejeon, Korea). It was titrated in Madin–Darby canine kidney (MDCK) cells to determine the 50% tissue culture infective dose (TCID50) by the Reed and Muench method [34]. MDCK cells were maintained in Dulbecco’s modified Eagle medium (DMEM; Therem Fischer Scientific, Waltham, MA, USA) containing 5% fetal bovine serum (FBS) (Sigma-Aldrich, St. Louis, MO. USA).

Sample solutions containing H1N1 at different concentrations (1–10^5^ TCID, tissue culture infective dose, 50/mL) were prepared in phosphate-buffered saline (PBS, pH 7.4) through 10-fold dilution. Their RNA was extracted using AccuPrep® Viral RNA Extraction Kit (Bioneer Co., Daejeon, Korea). Extracted RNA was amplified by quantitative reverse transcriptase-polymerase chain reaction (qRT-PCR) using a LightCycler® Nano (Roche, Basel, Switzerland). The primers used to amplify a 244-bp of *M* gene coding matrix in H1N1 were ATGAGYCTTYTAACCGAGGTCGAAACG for the forward primer and TGGACAAANCGTCTACGCTGCAG for the reverse primer [35]. The following conditions were used for qRT-PCR: isothermal incubation at 50 °C for 15 min, and 45 cycles of three temperatures (95 °C for 15 s, 60 °C for 10 s, and 72 °C for 30 s). A standard curve for H1N1 was generated by plotting quantification of cycle (Cq) versus the logarithm of the H1N1 concentrations.

### 2.2. Preparation of Antibody-Conjugated Magnetic Nanoparticles (Ab-MNPs) 

Amine-functionalized magnetic nanoparticles (MNPs) with 50 nm diameter (Chemicell Co., Berlin, Germany) were suspended in 2-(*N*-morpholino)ethanesulfonic acid (MES) buffer (200 mM, pH 6.0) and sonicated for 40 s to eliminate the aggregation of MNPs. A solution of the MNPs (1 mg/mL) in MES buffer was then made to react with glutaraldehyde (2.5% v/v) at room temperature (RT) for 1 h using a rotary incubator and then washed with borate buffer (10 mM, pH 8.4) to remove residual glutaraldehyde solution. MNPs functionalized with aldehyde were mixed with anti-hemagglutinin alpha-1 monoclonal antibody from KRIBB (50 μg/mL, final conc.) and incubated overnight at 4 °C. Ab-MNPs were washed with borate buffer and the free unreacted aldehyde was blocked with 1% bovine serum albumin (BSA) (Thermo Fisher Scientific) at 4 °C for 1 h. Borate buffer was used to wash and remove residual BSA. Sodium cyanoborohydride (20 mg/mL, final conc.) in borate buffer was used to treat the Ab-MNPs. Ab-MNPs were washed with Tris-HCl buffer (pH 8) and stored in PBS (pH 7.4) at 4 °C [16].

### 2.3. Microfabrication and Assembly of Microfluidic Preconcentration and Nucleic Amplification System (μFPNAS) 

It consists of three layers. All the three layers were individually designed in Inventor® professional (Autodesk Inc., Seoul, Korea) (Figure 1b). The top layer (lid layer) contained a chamber (60 mm × 20 mm) for flowing in sample solutions. The middle layer (concentration layer) contained a truncated trapezoidal-shaped preconcentration chamber (30 mm × 20 mm and 4 mm × 8 mm) for preconcentrating H1N1. The bottom layer (PCR layer) contained a channel (1 mm × 5 mm × 0.5 mm) and a PCR chamber (4 mm × 8 mm × 0.5 mm). 

For fabrication, the moulds for each layer were printed using a digital light-processing (DLP) 3D printer (Carima Co., Seoul, Korea). They were then exposed to ultraviolet (UV) light for 10 min to complete the crosslinking of the polymeric resin. The surface structure of each mould (Figure S1) was replicated onto a polydimethylsiloxane (PDMS) (Sylgard 184, Dow Corning, Midland, MI, USA) layer by soft lithography. The inlet and outlet holes were punched onto the first layer with a biopsy punch (1 mm). The layers were aligned and bonded to each other through oxygen plasma treatment (CUTE-MPR, Femto science, Hwasung, Korea) at 60 watts for 30 s. Finally, the channel and chamber surface were passivated with BSA by filling 100 μL of BSA (50 μg/mL) in PBS (pH 7.4) into the μFPNAS and incubating at 80 °C for 1 h to inhibit non-specific absorption of Ab-MNPs and MSBs into the PDMS surface [36].

### 2.4. On-Chip Preconcentration of H1N1 in Phosphate-Buffered Saline (PBS) and Saliva

Ten millilitres of PBS containing Ab-MNPs (10^12^ particles/mL) were mixed with 100 μL of PBS containing H1N1 at 10^4^ TCID50/mL in a 50 mL conical tube and incubated at 37 °C for 30 min before the preconcentration on μFPNAS.

Saliva was obtained from healthy volunteers according to the Institutional Review Board (IRB) of Sungkyunkwan University (SKKU) (approval number SKKU 2017-11-006). H1N1 (10^2^–10^4^ TCID50/mL, final conc.) was spiked into 1 mL saliva and the spiked sample was mixed with 9 mL of PBS containing Ab-MNPs (10^12^ particles/mL, final conc.). Then, the mixture was incubated at 37 °C for 30 min.

After the incubation, samples containing H1N1 were introduced into the μFPNAS at 2 mL/min through the inlet with the aid of a syringe pump (Harvard Apparatus, Holliston, MA, USA) while the neodymium magnet was located underneath the μFPNAS. After 5 min, only virus-Ab-MNPs complexes were preconcentrated in the chamber, while other waste molecules flowed through the outlet (Figure 1c). Once the preconcentration step was done, preconcentrated H1N1 in the μFPNAS was directly removed from the preconcentration chamber by a pipette and moved to a propylene tube (15 μL) after cutting the top lid layer with a razor blade. 2 μL of the preconcentrated sample in the tube was used for qRT-PCR to calculate virus-capturing efficiency and preconcentration fold. 

### 2.5. Virus-Capturing Efficiency

To calculate the relative capturing efficiency, we first obtained the standard curve of Cq values at concentrations (1–10^5^ TCID50/mL) of H1N1 in PBS. Using the standard curve, the Cq value of a preconcentrated sample by μFPNAS was converted to a virus concentration. Then, the relative capturing efficiency was calculated by the preconcentrated virus concentration in the μFPNAS divided by the theoretical virus concentration of the same sample when the virus in the sample was assumed to be 100% captured. 

Based on the relative capturing efficiency, its respective preconcentration fold was calculated using the following formula.

$$Preconcentration\;fold=\left(C.E.\right)\left(\frac{Vi}{Vp}\right)$$

where *C.E.* is the relative capturing efficiency, *Vi* and *Vp* are the volumes of the initial and preconcentrated samples.

### 2.6. Numerical Analysis of Heat Transfer in μFPNAS during Thermocycling

**Figure 2:**
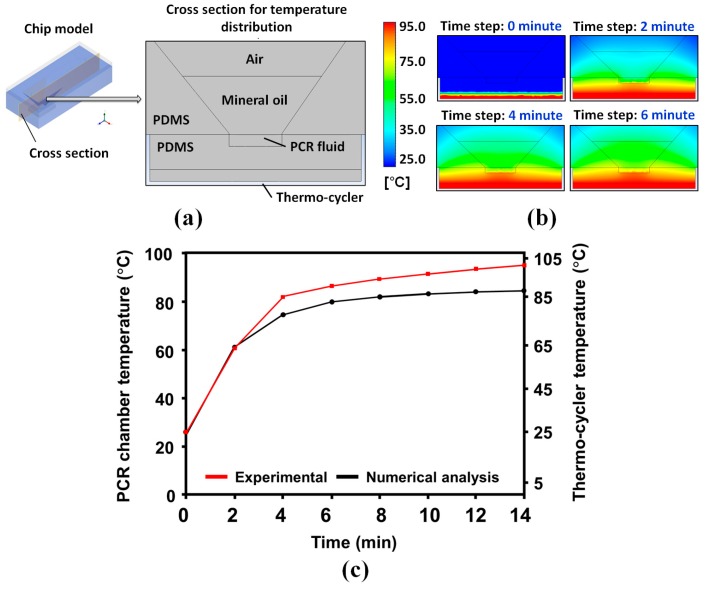
Heat-transfer profiles in the µFPNAS. (**a**) A cross section image of the µFPNAS. (**b**) Temperature changes in the PCR chamber for 6 min. (**c**) Comparison of experimental and numerical results. The numerical analysis was performed with ANSYS (Ansys Inc., Canonsburg, PA, USA).

Heat transfer in the μFPNAS during thermocycling was simulated with ANSYS Student Version (Ansys Inc., Canonsburg, PA, USA) to determine the temperature difference between the thermocycler and the PCR chamber as the thickness of the μFPNAS has an effect on heat transfer. For numerical analysis for heat transfer in the μFPNAS during thermocycling, the analysis domain for heat-transfer simulation was divided into four different sections; air, mineral oil, PCR fluid and PDMS (Figure 2a) and their own mechanical properties (molar mass, density, specific heat capacity and thermal conductivity) (Table S1) were individually set. Then, the simulation started with the application of heat at 95 °C from the bottom of the μFPNAS and the average temperature of PCR fluid was measured every 2 min for 14 min using a thermocouple during the experimental procedure. 

### 2.7. On-Chip Reverse Transcriptase-Polymerase Chain Reaction (RT-PCR) 

Once the virus preconcentration step was completed, a two-temperature PCR was performed with the integrated PCR system by adding 25 μL of PCR master mix to the PCR chamber via the PCR reagents inlet (Figure 1d). The PCR master mix containing 1× PCR buffer (20 mM Tris-HCl, pH 8.4, 50 mM KCl, 6 mM MgCl2), dNTPs (0.2 mM), and Taq DNA polymerase (5 U/μL) was purchased from Promega (Madison, WI, USA). The same primers for *M* gene used for qRT-PCR was used as well. To prevent evaporation of the PCR mixture, 200 μL of mineral oil was added to the chamber to cover PCR reagents during thermal cycling (Figure 1d). The μFPNAS was placed on a heat block (10 cm × 10 cm) modified from a C1000 Touch™ thermocycler (Bio-RAD, Hercules, CA, USA) (Figure S2). RT-PCR was performed by setting the temperature of the microchamber at the following temperatures for the respective time: 52 °C for 33 min for reverse transcription, 102 °C for 20 s for denaturation, 62.5 °C for 45 s for annealing and 75 °C for 40 s for extension. The three phases were repeated for 40 cycles and the final extension was at 75 °C for 5 min. The same primers were used as with the qRT-PCR.

RT-PCR products were confirmed by separating DNA in a 2% tris-acetate-EDTA (TAE) agarose gel at 100 V for 30 min. Agarose powder (DyneBio Inc., Seongnam, Korea) was mixed with 1× TAE buffer and heated in a microwave oven for 1 min. Gels were observed using UNOK-8000 electrophoresis gel documentation system (Korea Biotech, Daejeon, Korea).

### 2.8. Statistical Data Analysis

All the data displayed here was based on the mean ± standard deviation of three separate experiments. We used Student’s *t*-test to compare experiments performed in different settings. If the *p*-value is less than 0.05, it was considered significant.

## 3. Results

### 3.1. Simulation of Heat Transfer Profiles at PCR Chamber

Temperature changes of PCR fluid in the μFPNAS were analysed by graphically depicting temperature distributions on the cross-section of the analysis domain every 2 min (Figure 2a,b). The heat transfer tendency was confirmed by the comparison of experimental and numerical results (Figure 2c). The results showed that the heat was gradually transferred from the heat source to the PCR fluid. The temperature in the PCR chamber reached 50 °C, 60 °C, 72 °C and 95 °C, respectively, when the temperature of the heater was maintained at 52 °C, 62.5 °C, 75 °C and 102 °C, indicating that there were some temperature differences between the heater and PCR chamber, potentially due to the thickness of the PDMS. 

### 3.2. Effect of Sample Volume on Capturing Efficiency and Preconcentratin Fold

**Figure 3:**
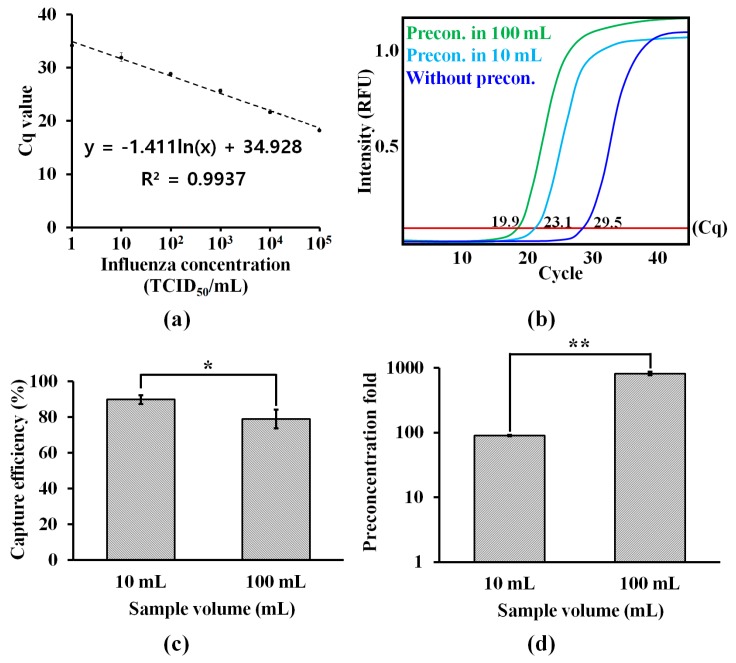
Quantitative reverse transcriptase-polymerase chain reaction (qRT-PCR) assays for evaluating the performance of the on-chip preconcentration. (**a**) A standard curve for H1N1 at different concentrations (1–10^5^ TCID50 (50% tissue culture infective dose)/mL) in phosphate-buffered saline (PBS). The Cq values plotted for each concentration are the mean of three replicates. (**b**) On-chip preconcentration of influenza virus H1N1 in different volume of samples containing 10^2^ TCID50/mL. (**c**) Effect of sample volume on viral capture efficiency. (**d**) Preconcentration fold. *: *p* < 0.05. **: *p* < 0.01. Student’s *t*-test. Sample number = 3.

**Figure 4:**
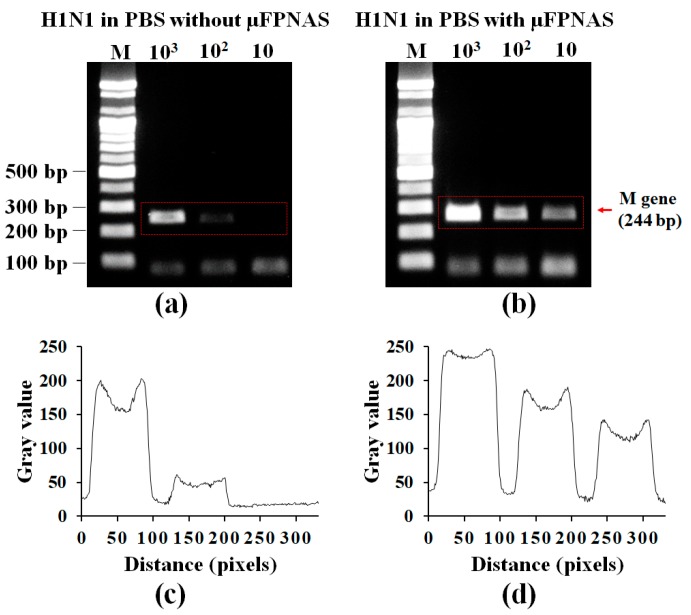
Validation of on-chip preconcentration and RT-PCR of H1N1 in PBS by gel electrophoresis. Gel electrophoresis of PCR products (*M* gene) from 1 mL PBS containing influenza A virus H1N1 at different concentration (10^2^–10^4^ TCID50/mL) without preconcentration (**a**) and with on-chip preconcentration and RT-PCR using µFPNAS. (**c**,**d**) Analysis of the intensity levels at the red rectangular areas in (**a**,**b**), respectively.

The standard curve for H1N1 (Figure 3a) showed that the Cq values of qRT-PCR for H1N1 were logarithmically dependent on the concentrations (1–10^5^ TCID50/mL). The curve was used to evaluate the effect of sample volumes on on-chip preconcentration. Without the on-chip preconcentration, its Cq value at 10^2^ TCID50/mL was 29.5 (Figure 3b). When H1N1, at the same concentration in different volumes (10 and 100 mL) of PBS, was preconcentrated using μFPNAS, their respective Cq values were reduced to 23.1 and 19.9, respectively. This showed that the on-chip preconcentration enhanced the amplification (Figure 3b) and this enhancement was dependent on the volume (Figure 3c). Although the sample volume negatively affected the virus-capturing efficiency, the capturing efficiency, even in 100 mL, was still maintained at about 80% (Figure 3c). When the data were converted into the preconcentration fold as shown in Figure 4d, the results indicate that the preconcentration fold increased as the sample volume increased.

### 3.3. On-Chip RT-PCR for Preconcentration of H1N1 Virus and Amplification of Target RNA in PBS

H1N1 RNA amplified by µFPNAS was confirmed by gel electrophoresis. This result showed that up to 10^2^ TCID50/mL was detectable in PBS (Figure 4b). As a result, the virus preconcentration and RT-PCR conducted on the µFPNAS improved the LOD by 10 orders of magnitude as compared to a non-treated sample (Figure 4a). The intensity levels of the gel electrophoresis was analysed with ImageJ (Figure 4c,d), showing an increase of the intensity level of the treated samples as compared to the untreated sample.

### 3.4. Spike Test in Saliva

**Figure 5:**
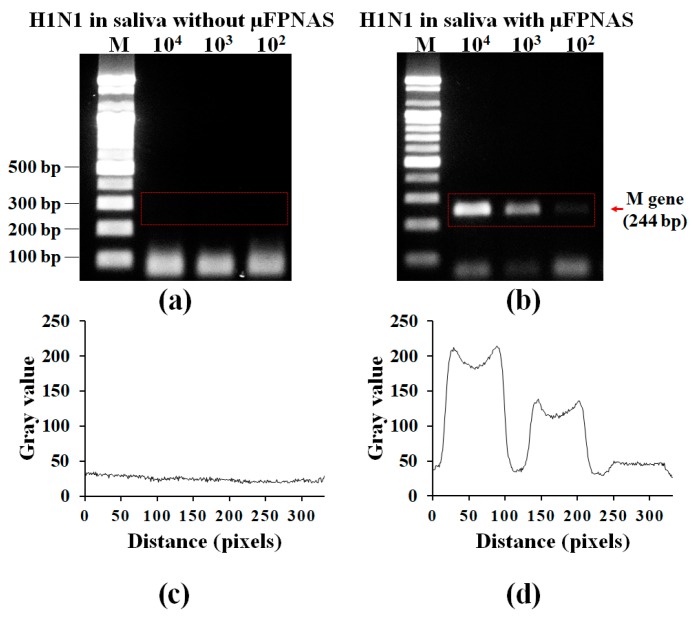
Validation of on-chip preconcentration and RT-PCR of H1N1 in saliva by gel electrophoresis. Gel electrophoresis of PCR products (*M* gene) from 1 mL saliva containing influenza A virus H1N1 at different concentration (10^2^–10^4^ TCID50/mL) without preconcentration (**a**) and on-chip preconcentration and RT-PCR using µFPNAS (**b**). (**c**,**d**) Analysis of intensity levels for the red rectangular areas in (**a**,**b**), respectively.

After preconcentration of 10^2^–10^4^ TCID50/mL influenza A virus H1N1 from 1 mL of saliva with the µFPNAS, RT-PCR was carried out. Figure 5c,d shows the analysis of the gel electrophoresis intensity levels by using ImageJ for the red rectangular area in Figure 5a,b respectively. It was possible to detect up to 100 TCID50/mL (Figure 5b). This is about 1000 times more sensitive than the results obtained from the sample without the use of the µFPNAS (Figure 5a).

## 4. Discussion

Previous reports have suggested that saliva inhibits PCR [17,18], resulting in decreased PCR activity. As a result, RNA extracted from viruses may be degraded by RNase. By separating and preconcentrating the target virus, PCR-inhibiting compounds were removed. The μFPNAS enabled effective separation and preconcentration of the target virus from the saliva sample, lowering the detection limit to 10^2^ TCID50/mL. Compared to the LOD of the non-treated sample, the pre-treated sample, shows that the LOD for the treated sample was improved 10 times in PBS and 1000 times in saliva. A microfluidic device for the preconcentration of a virus using Ab-MNPs and on-μFPNAS RT-PCR have been reported earlier [37]. This microfluidic device has a complicated structure, which makes the fabrication process difficult. However, our system has simple to fabricate preconcentration and amplification chambers capable of integrated preconcentration of virus and amplification of the target gene in the same device and the same location. Furthermore, our system minimized target loss due to movement of the preconcentrated sample from one chamber to other chambers. Unlike the previous published device handling 100 µL of sample, our device can handle a large volume (up to 100 mL) and obtain a higher preconcentration fold. The electromagnet used in the previous published device has a weaker magnetic force as compared to a permanent magnet, so the time required to preconcentrate the same amount of virus from the same volume sample is much longer. As a result, it cannot handle large volume samples. However, our system was able to preconcentrate 10 mL of sample in 5 min by flowing the sample at a higher flow rate (2 mL/min) and using a permanent magnet for the collection of the virus Ab-MNPs complex. The final volume of the virus Ab-MNP complex after preconcentration is approximately 2 µL, so the final concentration of the sample is 200 times that of the initial sample. The results confirm that influenza A virus H1N1 present at low concentrations in saliva cannot be detected directly by RT-PCR (Figure 5a). In the μFPNAS, the viruses were preconcentrated on a chip using Ab-MNPs while the RT-PCR was performed on the target gene of the preconcentrated virus. If further RNA extraction and purification steps are added to the μFPNAS, a more concentrated and pure RNA can be obtained, which might improve the detection limit even further. The primer used for the detection of influenza A virus H1N1 is based on the *M* gene (244 bp), but the use of primer that is based on the hemagglutinin is required for the identification of their subtypes.

The thermocycler (Figure S2) for RT-PCR in our microfluidic device is too large to be used for detection of the virus in situ. By replacing it with a commercially available palm-size thermocycler, our microfluidic system could be used for detection of influenza A virus H1N1 in situ. 

## 5. Conclusions

In this study, we have developed a microfluidic device, the µFPNAS, consisting of a trapezoidal preconcentration microchamber and a RT-PCR chamber. This device improved the LOD for molecular detection of influenza A virus H1N1 and reduced the sample pre-treatment time required for the detection of H1N1 virus from saliva. Since the requirement for specialized equipment was reduced by the simple operation of the microfluidic device, it can be used as a point-of-care detection device. Furthermore, the microfluidic device was capable of preconcentrating a large volume sample (up to 100 mL) within 2 hours. As a result, the limit of detection was improved. Automation of the µFPNAS-based system can provide a powerful tool for the rapid diagnostic of infectious diseases such as influenza A virus H1N1 and as a quantitative virion analyser if integrated with optical sensors.

## Supplementary Materials

The following are available online at https://www.mdpi.com/2072-666X/11/2/203/s1: Figure S1: The 3D design (a, c, e) and their respective photographic images (b, d, f) of the master moulds for each layer: the lid (a, b), concentration (c, d), and PCR (e, f) layers., Figure S2: A tailor-made flat heat block was used to conduct chip-based PCR, instead of using conventional heat block which contains either 48 or 96 wells to hold PCR tubes, Table S1: Material properties used in numerical analysis.

## References

[1] Taubenberger J.K., Morens D.M. (2008). The pathology of influenza virus infections. Annu. Rev. Pathol.-Mech..

[2] Camp J.V., Bagci U., Chu Y.-K., Squier B., Fraig M., Uriarte S.M., Guo H., Mollura D.J., Jonsson C.B. (2015). Lower respiratory tract infection of the ferret by 2009 H1N1 pandemic influenza A virus triggers biphasic, systemic, and local recruitment of neutrophils. J. Virol..

[3] Dziąbowska K., Czaczyk E., Nidzworski D. (2018). Detection methods of human and animal influenza virus—current trends. Biosensors.

[4] Gatherer D. (2009). The 2009 H1N1 influenza outbreak in its historical context. J. Clin. Virol..

[5] Herrmann B., Larsson C., Zweygberg B.W. (2001). Simultaneous detection and typing of influenza viruses A and B by a nested reverse transcription-PCR: Comparison to virus isolation and antigen detection by immunofluorescence and optical immunoassay (FLU OIA). J. Clin. Microbiol..

[6] Pachucki C.T., Creticos C. (1988). Early detection of influenza virus by using a fluorometric assay of infected tissue culture. J. Clin. Microbiol..

[7] Fouchier R.A., Bestebroer T.M., Herfst S., Van Der Kemp L., Rimmelzwaan G.F., Osterhaus A.D. (2000). Detection of influenza A viruses from different species by PCR amplification of conserved sequences in the matrix gene. J. Clin. Microbiol..

[8] Choi Y., Goyal S.M., Kang S., Farnham M., Joo H. (2002). Detection and subtyping of swine influenza H1N1, H1N2 and H3N2 viruses in clinical samples using two multiplex RT-PCR assays. J. Virol. Methods.

[9] Ito M., Watanabe M., Nakagawa N., Ihara T., Okuno Y. (2006). Rapid detection and typing of influenza A and B by loop-mediated isothermal amplification: Comparison with immunochromatography and virus isolation. J. Virol. Methods.

[10] Shan S., Ko L.-S., Collins R.A., Wu Z., Chen J., Chan K.-Y., Xing J., Lau L.-T., Yu A.C.-H. (2003). Comparison of nucleic acid-based detection of avian influenza H5N1 with virus isolation. Biochem. Biophys. Res. Commun..

[11] Lv J., Wei B., Chai T., Xia X., Miao Z., Yao M., Gao Y., Huang R., Yang H., Roesler U. (2011). Development of a real-time RT-PCR method for rapid detection of H9 avian influenza virus in the air. Arch. Virol..

[12] Cuchacovich R. (2006). Clinical applications of the polymerase chain reaction: An update. Infect. Dis. Clin. North Am..

[13] Yang S., Rothman R.E. (2004). PCR-based diagnostics for infectious diseases: Uses, limitations, and future applications in acute-care settings. Lancet Infect. Dis..

[14] Johnson S.R., Martin D.H., Cammarata C., Morse S.A. (1995). Alterations in sample preparation increase sensitivity of PCR assay for diagnosis of chancroid. J. Clin. Microbiol..

[15] Amicosante M., Richeldi L., Trenti G., Paone G., Campa M., Bisetti A., Saltini C. (1995). Inactivation of polymerase inhibitors for Mycobacterium tuberculosis DNA amplification in sputum by using capture resin. J. Clin. Microbiol..

[16] Kim Y., Lee J., Park S. (2018). A 3D-printed millifluidic platform enabling bacterial preconcentration and DNA purification for molecular detection of pathogens in blood. Micromachines.

[17] Mättö J., Saarela M., Alaluusua S., Oja V., Jousimies-Somer H., Asikainen S. (1998). Detection of Porphyromonas gingivalisfrom saliva by PCR by using a simple sample-processing method. J. Clin. Microbiol..

[18] Ochert A., Boulter A., Birnbaum W., Johnson N., Teo C. (1994). Inhibitory effect of salivary fluids on PCR: Potency and removal. PCR Methods Appl..

[19] Islam M.A., Heuvelink A.E., Talukder K.A., Zwietering M.H., De Boer E. (2006). Evaluation of immunomagnetic separation and PCR for the detection of *Escherichia coli* O157 in animal feces and meats. J. Food Prot..

[20] Ganesh I., Tran B.M., Kim Y., Kim J., Cheng H., Lee N.Y., Park S. (2016). An integrated microfluidic PCR system with immunomagnetic nanoparticles for the detection of bacterial pathogens. Biomed. Microdevices.

[21] Zhu P., Shelton D.R., Li S., Adams D.L., Karns J.S., Amstutz P., Tang C.-M. (2011). Detection of *E. coli* O157: H7 by immunomagnetic separation coupled with fluorescence immunoassay. Biosens. Bioelectron..

[22] Kabir S. (2004). Detection of Helicobacter pylori DNA in feces and saliva by polymerase chain reaction: A review. Helicobacter.

[23] Song J., Mauk M.G., Hackett B.A., Cherry S., Bau H.H., Liu C. (2016). Instrument-free point-of-care molecular detection of Zika virus. Anal. Chem..

[24] Niemz A., Ferguson T.M., Boyle D.S. (2011). Point-of-care nucleic acid testing for infectious diseases. Trends Biotechnol..

[25] Enroth H., Engstrand L. (1995). Immunomagnetic separation and PCR for detection of *Helicobacter pylori* in water and stool specimens. J. Clin. Microbiol..

[26] Chen J., Shi X., Gehring A.G., Paoli G.C. (2014). Automated immunomagnetic separation for the detection of *Escherichia coli* O157: H7 from spinach. Int. J. Food Microbiol..

[27] Luppa P.B., Müller C., Schlichtiger A., Schlebusch H. (2011). Point-of-care testing (POCT): Current techniques and future perspectives. Trends Anal. Chem..

[28] Bu M., Christensen T.B., Smistrup K., Wolff A., Hansen M.F. (2008). Characterization of a microfluidic magnetic bead separator for high-throughput applications. Sens. Actuators A-Phys..

[29] Fedio W.M., Jinneman K.C., Yoshitomi K.J., Zapata R., Wendakoon C.N., Browning P., Weagant S.D. (2011). Detection of *E. coli* O157: H7 in raw ground beef by Pathatrix™ immunomagnetic-separation, real-time PCR and cultural methods. Int. J. Food Microbiol..

[30] Pamme N. (2006). Magnetism and microfluidics. Lab Chip.

[31] Roda A., Mirasoli M., Roda B., Bonvicini F., Colliva C., Reschiglian P. (2012). Recent developments in rapid multiplexed bioanalytical methods for foodborne pathogenic bacteria detection. Mikrochim. Acta.

[32] Xia N., Hunt T.P., Mayers B.T., Alsberg E., Whitesides G.M., Westervelt R.M., Ingber D.E. (2006). Combined microfluidic-micromagnetic separation of living cells in continuous flow. Biomed. Microdevices.

[33] Yang L., Banada P.P., Chatni M.R., Lim K.S., Bhunia A.K., Ladisch M., Bashir R. (2006). A multifunctional micro-fluidic system for dielectrophoretic concentration coupled with immuno-capture of low numbers of *Listeria monocytogenes*. Lab Chip.

[34] Reed L.J., Muench H. (1938). A simple method of estimating fifty per cent endpoints. Am. J. Epidemiol..

[35] Fujitsuka A., Tsukagoshi H., Arakawa M., Goto-Sugai K., Ryo A., Okayama Y., Mizuta K., Nishina A., Yoshizumi M., Kaburagi Y. (2011). A molecular epidemiological study of respiratory viruses detected in Japanese children with acute wheezing illness. BMC Infect. Dis..

[36] Besecker J., Cornell K.A., Hampikian G. (2013). Dynamic passivation with BSA overcomes LTCC mediated inhibition of PCR. Sens. Actuators B Chem..

[37] Lien K.-Y., Lin J.-L., Liu C.-Y., Lei H.-Y., Lee G.-B. (2007). Purification and enrichment of virus samples utilizing magnetic beads on a microfluidic system. Lab Chip.

