# Supplementary material for: Integrated Microfluidic Preconcentration and Nucleic Amplification System for Detection of Influenza A Virus H1N1 in Saliva
Source: Micromachines (Basel). 2020 Feb 16;11(2):203. doi: 10.3390/mi11020203 (PMC7074655; doi:10.3390/mi11020203)
Supplement: Supplementary file 1 [file micromachines-11-00203-s001.pdf]

Supplementary Materials: Integrated Microfluidic Preconcentration and Nucleic Amplification System for Detection of Influenza a Virus H1N1 in Saliva

Yonghee Kim, Abdurhaman Teyib Abafogi, Buu Minh Tran, Jaewon Kim, Jinyeop Lee, Zhenzhong Chen, Pan Kee Bae, Kyoungsook Park, Yong-Beom Shin, Danny van Noort, Nae Yoon Lee and Sungsu Park

Table 1. Material properties used in numerical analysis.

Materials	Molar Mass (g/mol)	Density (kg/m³)	Specific Heat Capacity (J/kg·K)	Thermal Conductivity (W/m·K)
Air	28.96	1.275	1.0044×10^3	2.61×10^{-2}
PCR fluid (Water)	10.82	997	4181.7	0.6069
PDMS (10:1)	1250	980	1100	0.18
Mineral oil	480	850	1670	0.14

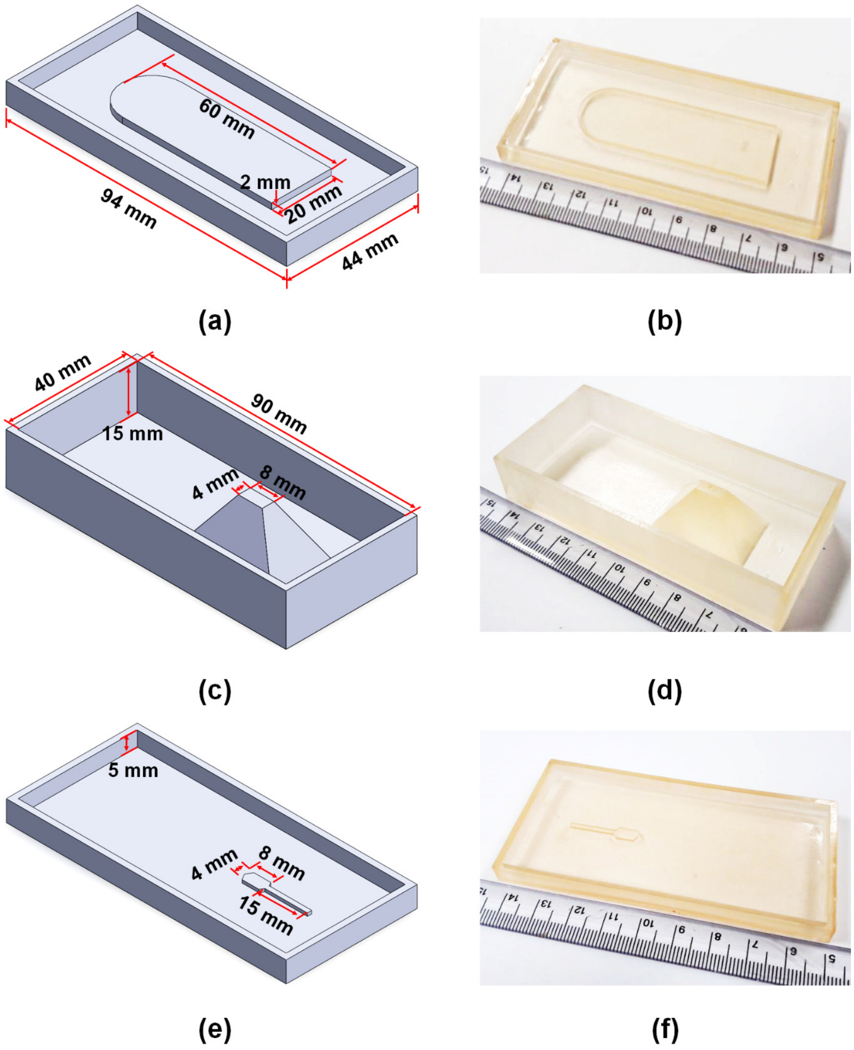

Figure S1. The 3D design (**a**, **c**, **e**) and their respective photographic images (**b**, **d**, **f**) of the master moulds for each layer: the lid (**a**, **b**), concentration (**c**, **d**), and PCR (**e**, **f**) layers.

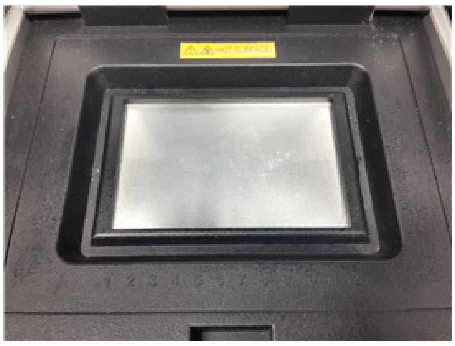
.

Figure S2. A tailor-made flat heat block was used to conduct chip-based PCR, instead of using conventional heat block which contains either 48 or 96 wells to hold PCR tubes,
